# Supplementary material for: A targetable CoQ-FSP1 axis drives ferroptosis- and radiation-resistance in KEAP1 inactive lung cancers
Source: Nat Commun. 2022 Apr 22;13:2206. doi: 10.1038/s41467-022-29905-1 (PMC9033817; doi:10.1038/s41467-022-29905-1)
Supplement: Supplementary file 1 — Supplementary Information [file 41467_2022_29905_MOESM1_ESM.pdf]

Supplementary Information

A targetable CoQ-FSP1 axis drives ferroptosis- and radiation-resistance in KEAP1 inactive lung cancers

Pranavi Koppula, Guang Lei, Yilei Zhang, Yuelong Yan, Chao Mao, Lavanya Kondiparthi, Jiejun Shi, Xiaoguang Liu, Amber Horbath, Molina Das, Wei Li, Masha V. Poyurovsky, Kellen Olszewski, Boyi Gan

Supplementary Figure 1

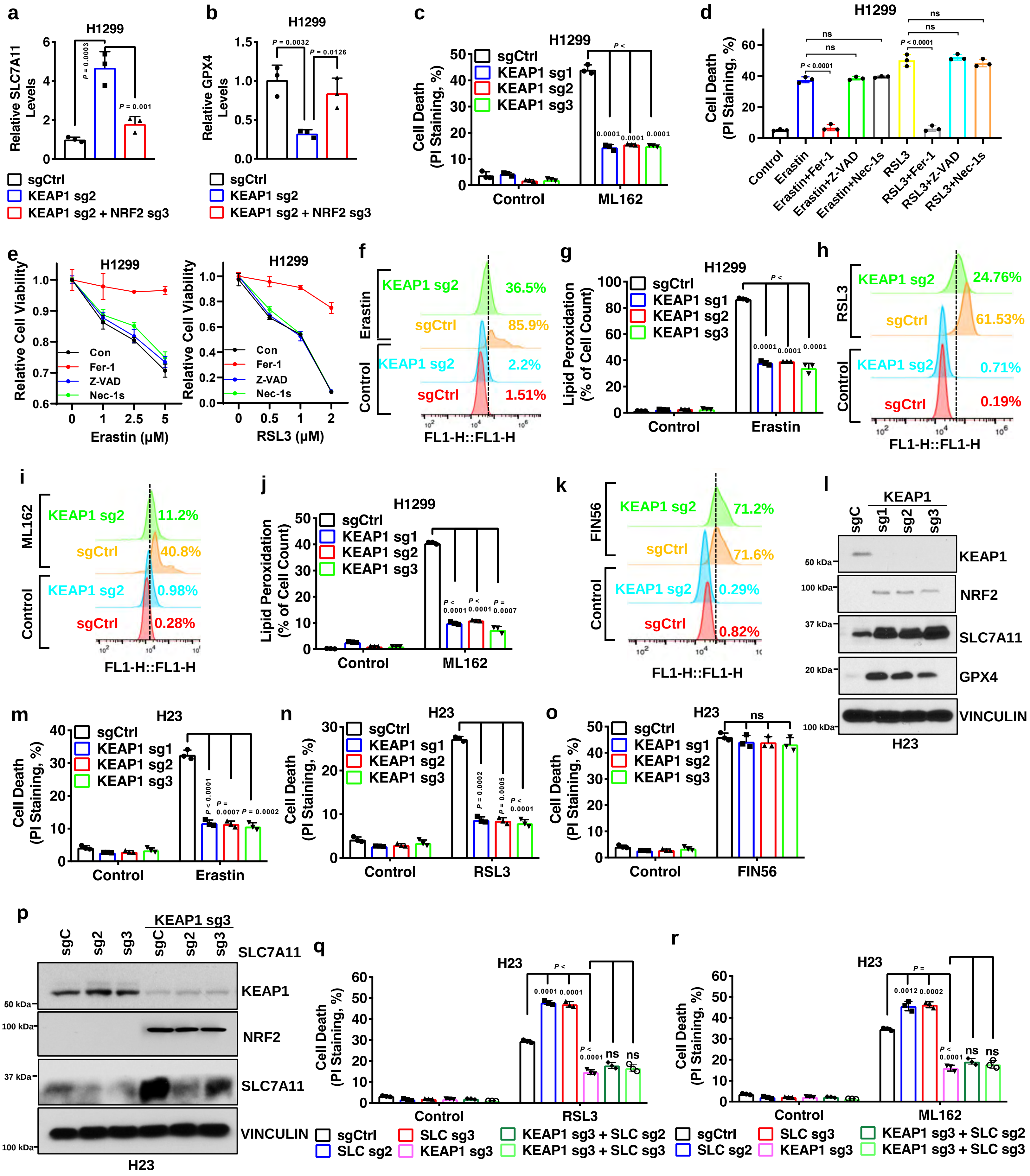

Supplementary Figure 1. KEAP1 regulates ferroptosis in a SLC7A11-independent manner in lung cancer cells.

a, b qRT-PCR analysis of *SLC7A11* (**a**) and *GPX4* (**b**) in *KEAP1/ NRF2* DKO H1299 cells. **c** Cell death upon ML162 treatment in *KEAP1* KO H1299 cells was analyzed by PI staining. **d, e** Cell death (**d**) and cell viability (**e**) measurement in *KEAP1* KO H1299 cells with indicated treatments. **f-k** Lipid peroxidation levels were determined for *KEAP1* KO H1299 cells treated with erastin (**f, g**), RSL3 (**h**), ML162, (**i, j**) and FIN56 (**k**). **l** Protein levels of KEAP1, NRF2, SLC7A11, GPX4 in *KEAP1* KO H23 cells were determined by western blotting. **m-o** Cell death upon erastin (**m**), RSL3 (**n**), or FIN56 (**o**) treatment in *KEAP1* KO H23 cells was analyzed by PI staining. **p** Protein levels of KEAP1, NRF2, SLC7A11 in *SLC7A11* KO, *KEAP1* KO and *SLC7A11/ KEAP1* DKO H23 cells. **q, r** Cell death was quantified by PI staining for H23 cells by RSL3 (**q**) or ML162 (**r**). Error bars are (if mentioned otherwise) mean +/- SD; *n* =3. P value was determined by two-tailed unpaired Student's t test (1a, b, d) and 2-way ANOVA (1c, 1g, 1j, 1m-o, 1q-r); ns, not significant. Source data are provided as a Source Data file.

Supplementary Figure 2

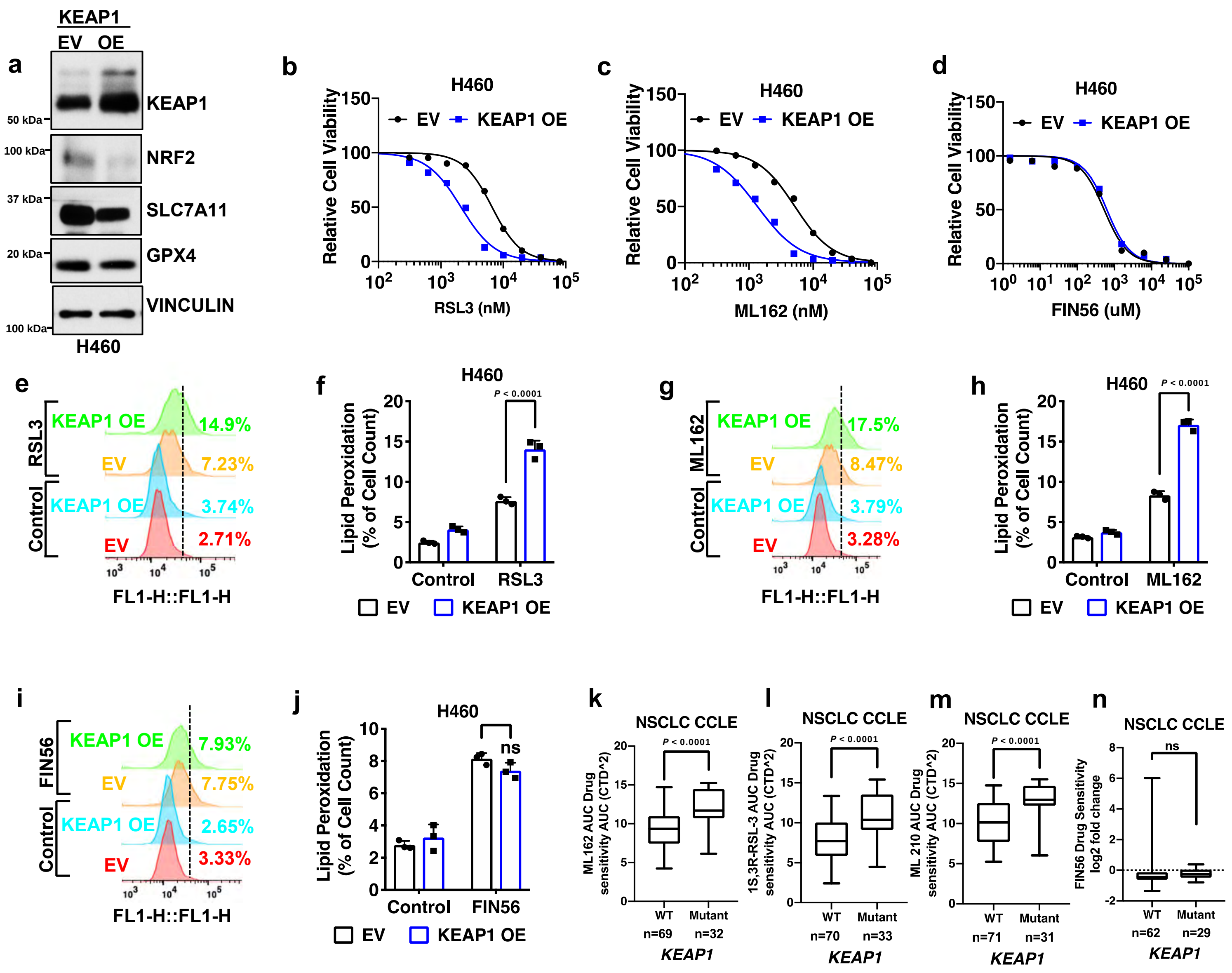

Supplementary Figure 2. KEAP1 regulates ferroptosis in lung cancer cells.

a Protein levels of KEAP1, NRF2, SLC7A11, GPX4 in H460 cells with KEAP1 overexpression (OE). **b-d** Cell viability was quantified by CCK8 for H460 cells by RSL3 (**b**), ML162 (**c**) and FIN56 (**d**). **e-j** Lipid peroxidation levels were determined for KEAP1 OE H460 cells treated with RSL3 (**e, f**), ML162 (**g, h**), or FIN56 (**i, j**). **k-n** *KEAP1* mutation correlates with resistance to ML162 (**k**), RSL3 (**l**), and ML210 (**m**), but not FIN56 (**n**). NSCLC: Non-Small Cell Lung Cancer; CCLE: Cancer Cell Line Encyclopedia. Error bars are (if mentioned otherwise) mean +/- SD; *n* =3. P value was determined by two-tailed unpaired Student's t test. Box and whisker plots show centre line at median, box limits at 25th/75th centiles and whiskers ±1.5× interquartile range (IQR); ns, not significant. Source data are provided as a Source Data file.

Supplementary Figure 3

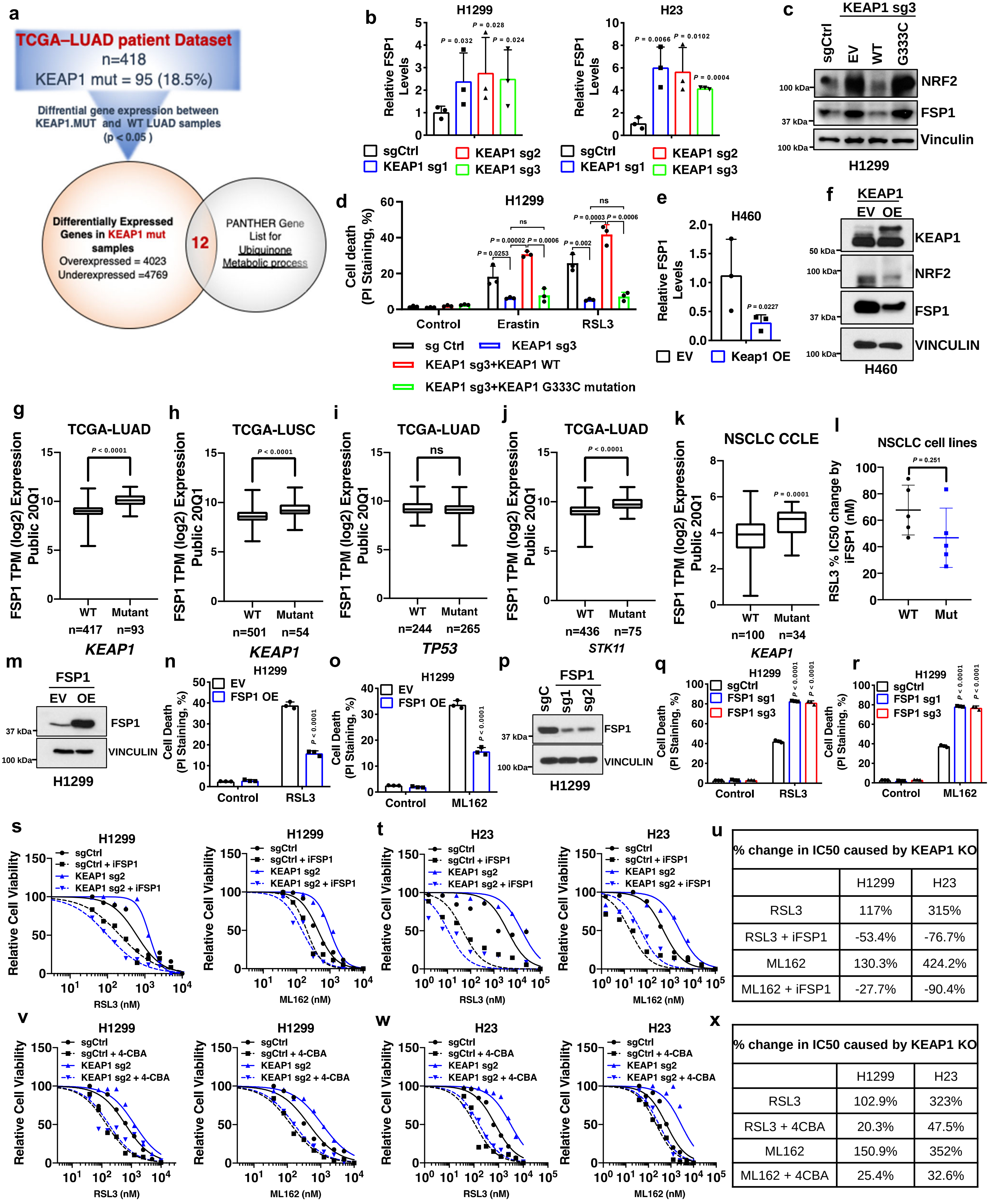

u

% change in IC50 caused by KEAP1 KO		
	H1299	H23
RSL3	117%	315%
RSL3 + iFSP1	-53.4%	-76.7%
ML162	130.3%	424.2%
ML162 + iFSP1	-27.7%	-90.4%

x

% change in IC50 caused by KEAP1 KO		
	H1299	H23
RSL3	102.9%	323%
RSL3 + 4CBA	20.3%	47.5%
ML162	150.9%	352%
ML162 + 4CBA	25.4%	32.6%

Supplementary Figure 3. KEAP1 regulates ferroptosis sensitivity through FSP1.

a Schematic of gene ontology analysis on overexpressed genes in *KEAP1* mutant LUAD. **b** qRT-PCR analysis of *FSP1* in *KEAP1* KO H1299 or H23 cells. **c** Protein levels of NRF2 and FSP1 in H1299 control cells or in H1299 *KEAP1* KO cells expressing empty vector (EV), KEAP1 wild-type (WT), or KEAP1 G333C mutant. **d** Cell death analysis of indicated H1299 cells treated with erastin or RSL3. **e** qRT-PCR analysis of *FSP1* in H460 cells with KEAP1 overexpression (OE). **f** Protein levels of KEAP1, NRF2, and FSP1 in KEAP1 OE H460 cells. **g-k** Correlation analysis of FSP1 expression levels in *KEAP1* mutant TCGA-LUAD (**g**), *KEAP1* mutant TCGA-LUSC (**h**), *p53* mutant TCGA-LUAD (**i**), *LKBI* mutant TCGA-LUAD (**j**), or *KEAP1* mutant NSCLC cell lines from CCLE datasets (**k**). TCGA: The Cancer Genome Atlas. **l** Percentage changes in RSL3 IC50 with iFSP1 cotreatment in NSCLC cell lines. **m** protein levels of FSP1 in H1299 cells with FSP1 OE. **n, o** Cell death analysis of H1299 cells with FSP1 OE treated with RSL3 (**n**) and ML162 (**o**). **p** Protein levels of FSP1 in *FSP1* KO H1299 cells. **q, r** Cell death analysis of *FSP1* KO H1299 cells treated with RSL3 (**q**) or ML162 (**r**). **s, t** Cell viability was quantified by CCK8 in *KEAP1* KO H1299 cells with iFSP1 treatment combined with RSL3 or ML162 (**s**); *KEAP1* KO H23 cells with iFSP1 treatment combined with RSL3 or ML162 (**t**). **u** Percentage changes in IC50 caused by *KEAP1* KO in H1299 and H23 cell lines upon the indicated treatments. **v, w** Cell viability was quantified by CCK8 in *KEAP1* KO H1299 cells with 4-CBA treatment combined with RSL3 or ML162 (**v**); *KEAP1* KO H23 cells with 4-CBA treatment combined with RSL3 or ML162 (**w**). **x** Percentage changes in IC50 caused by *KEAP1* KO in H1299 and H23 cell lines upon the indicated treatments. Error bars are (if mentioned otherwise) mean +/- SD; *n* =3. P value was determined by two-tailed unpaired Student's t test. Box and whisker plots show centre line at median, box limits at 25th/75th centiles and whiskers ±1.5× interquartile range (IQR); ns, not significant. Source data are provided as a Source Data file.

Supplementary Figure 4

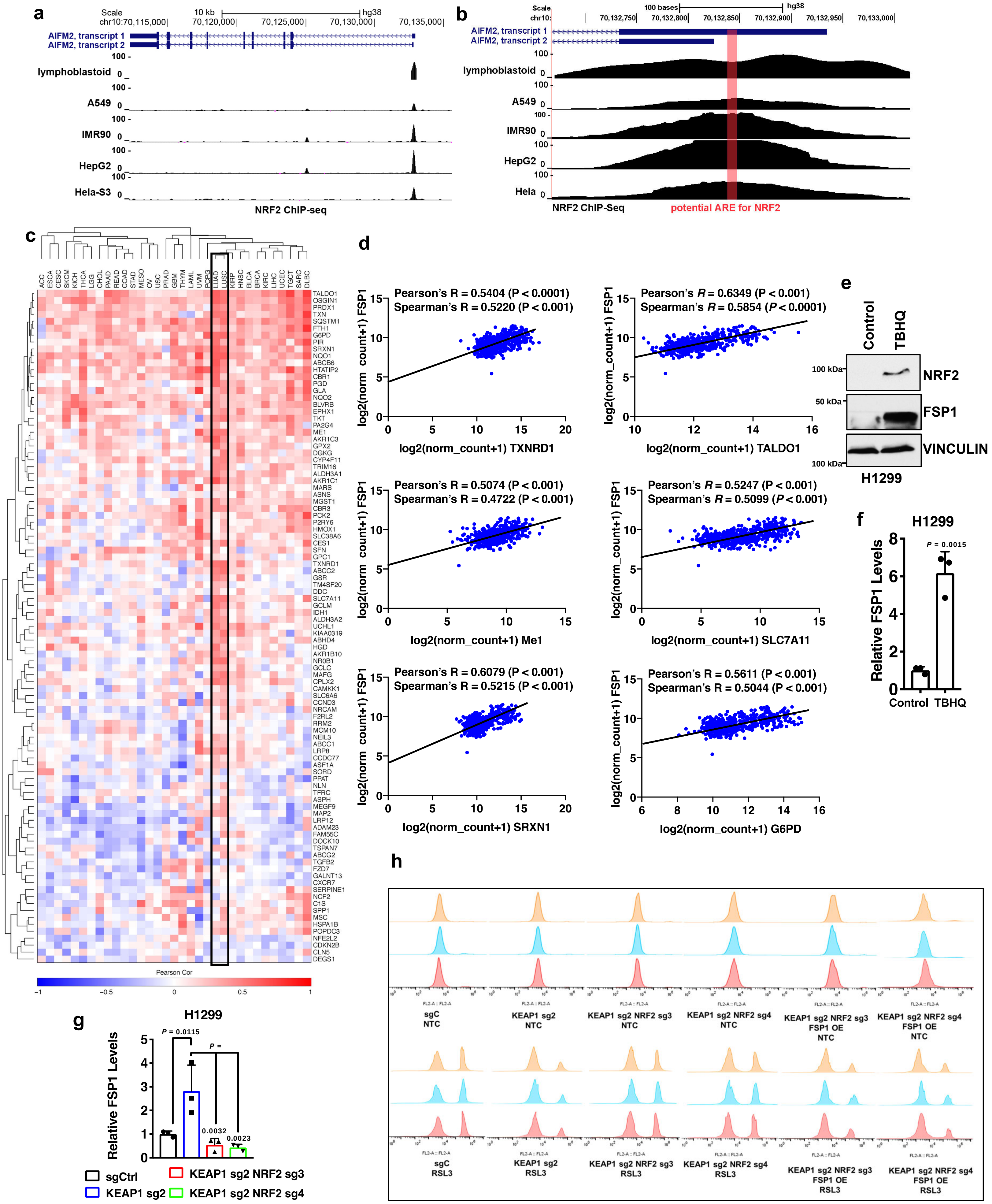

Supplementary Figure 4. KEAP1 regulates FSP1 through NRF2-mediated transcription.

a, **b** NRF2 ChIP-seq profiles in lymphoblastoid, A549, IMR90, HepG2 and Hela S3 cell lines (GEO datasets: GSE37589, GSE91894, GES91565, GSE91809 and GSE91997). **c** Unsupervised clustering analyses of FSP1 and NRF2 target genes in multiple cancers. **d** Correlation analyses of FSP1 and classical NRF2 target genes in TCGA-LUAD. **e** Protein levels of FSP1 and NRF2 in H1299 cells treated with TBHQ. **f** qRT-PCR analysis of *FSP1* in H1299 treated with TBHQ. **g** qRT-PCR analysis of *FSP1* in *KEAP1 NRF2* DKO H1299 cells. **h** FACS panels of PI staining from three independent repeats showing cell death upon RSL3 treatment in *KEAP1 NRF2* DKO H1299 cells overexpressing FSP1. For **f** and **j**, P value was determined by two-tailed unpaired Student's t test. Error bars are (if mentioned otherwise) mean +/- SD; *n* =3; ns, not significant. Source data are provided as a Source Data file.

Supplementary Figure 5

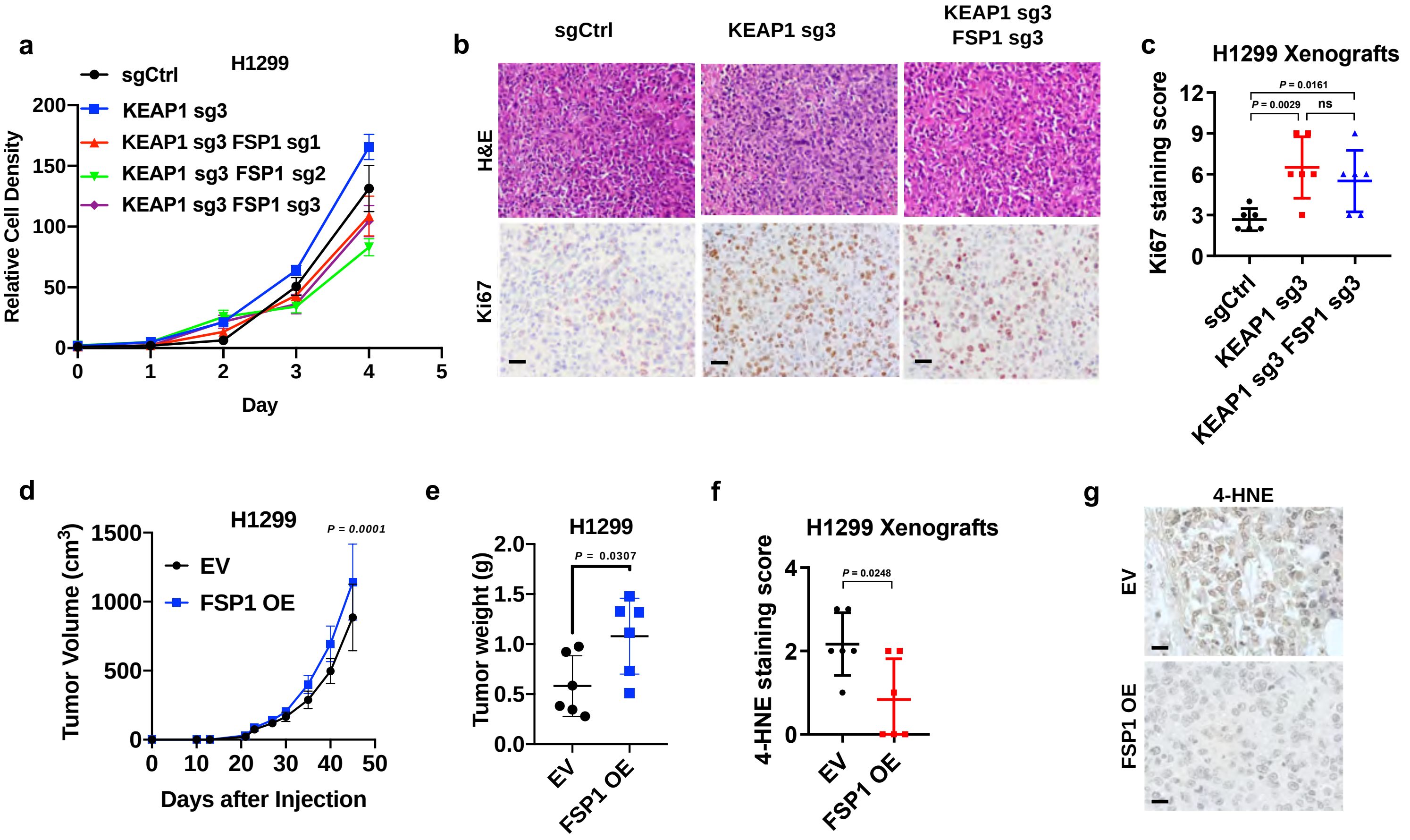

Supplementary Figure 5. FSP1 promotes tumorigenesis in lung cancer upon *KEAP1* deletion

a Proliferation rates of *KEAP1 FSP1* DKO H1299 cell lines. Error bars are means +/- SD, *n* = 3. **b** Representative images of hematoxylin and eosin and Ki67 immunohistochemical staining in *KEAP1 FSP1* DKO H1299 xenograft tumors. Scale bars, 50 µm. **c** Immunohistochemistry scoring of Ki67 staining in *KEAP1 FSP1* DKO H1299 xenograft tumor sections. Error bars are means +/- SD, *n* = 6 randomly selected magnification fields. **d** Tumor volumes of empty vector (EV) and FSP1-overexpressing (OE) H1299 xenograft tumors. Error bars are mean +/− SD; *n* = 6 tumors. **e** End point weights of EV and FSP1 OE H1299 xenograft tumors. Error bars are mean +/− SD; *n* = 6 tumors. **f, g** Immunochemistry staining scoring (**f**) and representative images (**g**; scale bars, 20 µm) of 4-HNE in indicated H1299 xenograft tumor sections. Error bars are means +/- SD, *n* = 6 randomly selected magnification fields. Data are presented as (if mentioned otherwise) mean +/- SD; *n* =3. P value was determined by two-tailed unpaired Student's t test; ns, not significant. Source data are provided as a Source Data file.

Supplementary Figure 6

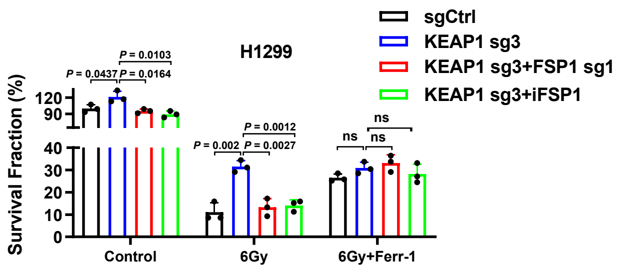

Supplementary Figure 6. FSP1 inhibition sensitizes *KEAP1* deficient lung cancer cells to radiation through ferroptosis.

Survival fraction of *KEAP1* KO H1299 cells with genetic or pharmacological inhibition of FSP1 treated with 6 Gy X-ray irradiation alone or with ferrostatin-1 pretreatment for 24h. Data are presented as (if mentioned otherwise) mean +/- SD; *n* =3. P value was determined by two-tailed unpaired Student's t test; ns, not significant. Source data are provided as a Source Data file.

Supplementary Figure 7

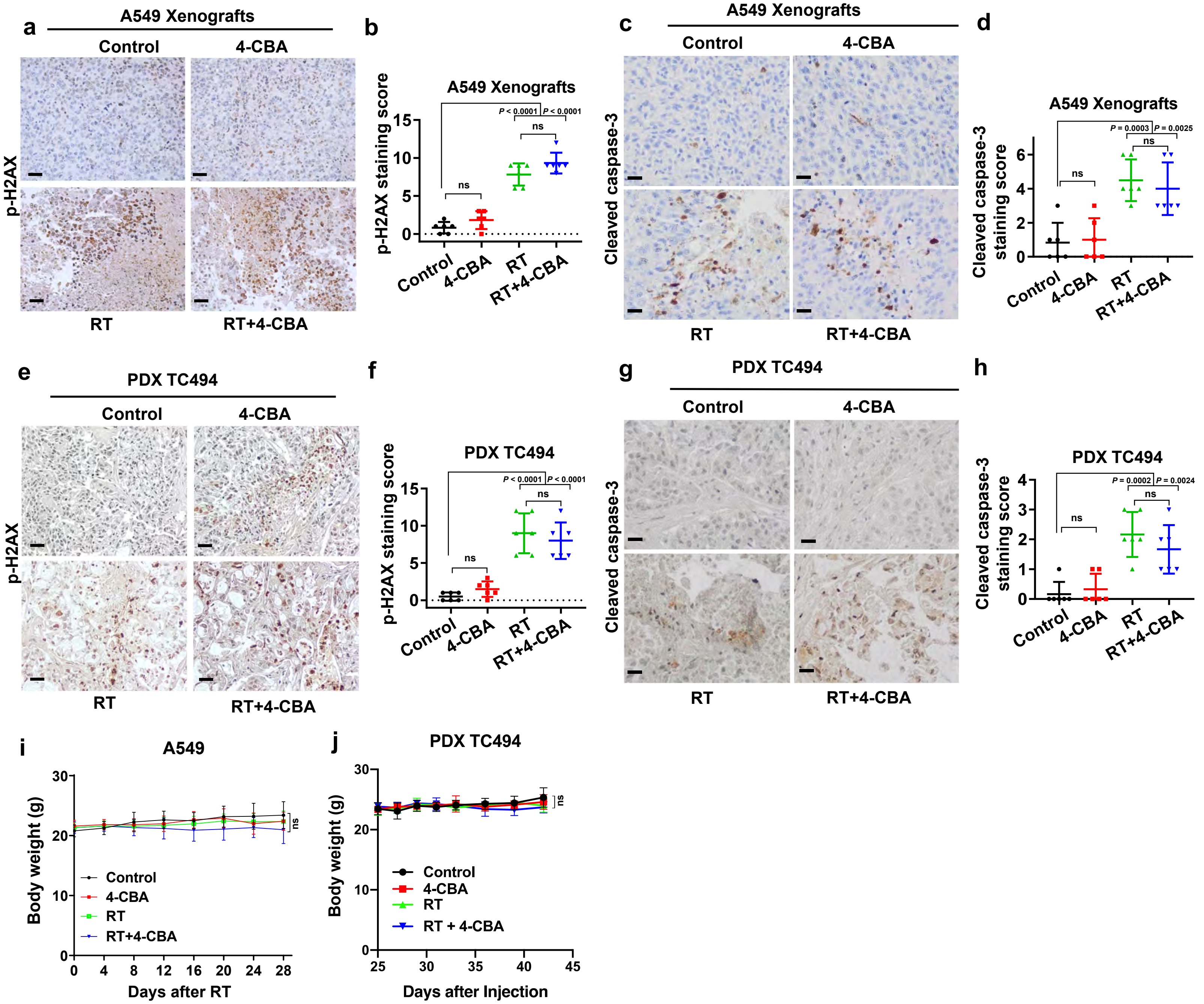

Supplementary Figure 7. Inhibiting CoQ synthesis reverses radioresistance in *KEAP1* deficient or mutant lung cancers.

a, b Representative images (**a**; scale bars, 20 μm) and scores (**b**) of IHC staining for p-H2AX in A549 xenograft tumors with indicated treatments. Error bars are means +/- SD, *n* = 6 randomly selected magnification fields. **c, d** Representative images (**c**; scale bars, 20 μm) and scores (**d**) of IHC staining for cleaved caspase-3 in A549 xenograft tumors with indicated treatments. Error bars are means +/- SD, *n* = 6 randomly selected magnification fields. **e, f** Representative images (**e**; scale bars, 20 μm) and scores (**f**) of IHC staining for p-H2AX in PDX TC494 tumors with indicated treatments. Error bars are means +/- SD, *n* = 6 randomly selected magnification fields. **g, h** Representative images (**g**; scale bars, 20 μm) and scores (**h**) of IHC staining for cleaved caspase-3 in PDX TC494 tumors with indicated treatments. Error bars are means +/- SD, *n* = 6 randomly selected magnification fields. **i, j** Mice weights of xenografts derived from A549 (**i**) or PDX TC494 tumors (**j**) grown at different time points treated with either 10 Gy X-ray irradiation or 4-CBA alone, or combined treatement of radiation and 4-CBA. Error bars are means +/- SD, *n* = 5-8 mice. P value was determined by two-tailed unpaired Student's t test; ns, not significant. Source data are provided as a Source Data file.

Supplementary Figure 8

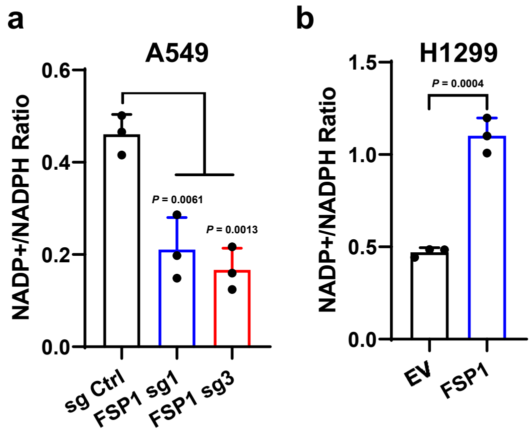

Supplementary Figure 8. Inhibiting CoQ synthesis reverses radioresistance in *KEAP1* deficient or mutant lung cancer.

a, b $NADP^+$/NADPH ratio in A549 *FSP1* KO cells (**a**) and H1299 *FSP1* overexpressing cells (**b**). Error bars are presented as mean +/- SD; *n* =3 independent samples. P value was determined by two-tailed unpaired Student's t test. Source data are provided as a Source Data file.

Supplementary Figure 9

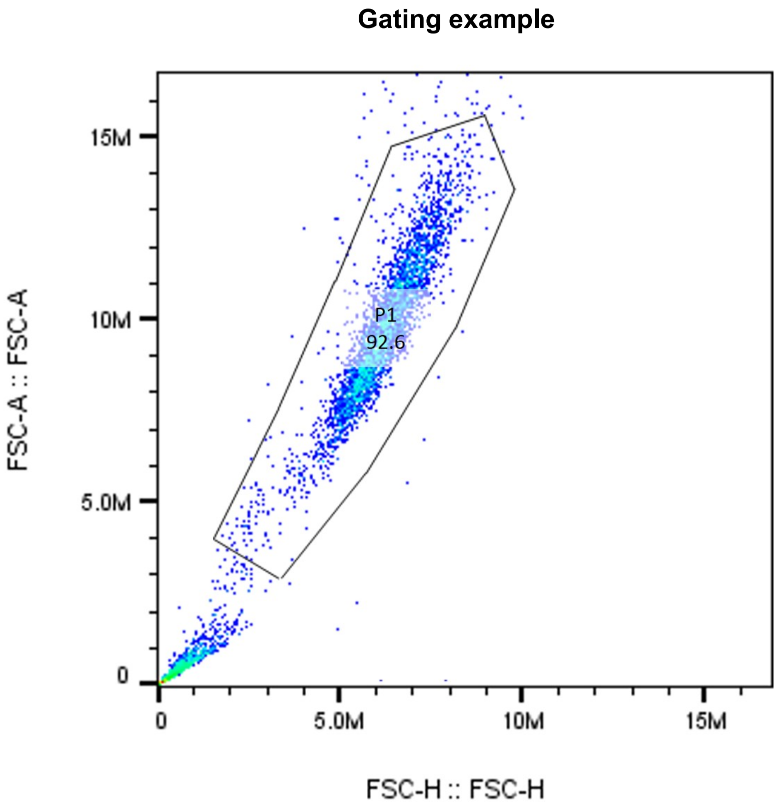

Supplementary Figure 9. Gating strategies for lipid peroxidation analysis through flow cytometry.

Supplementary Table 1. Ubiquinone metabolic pathway enrichment

Ubiquinone metabolic pathway enrichment		
	log2FC	p value
COQ6	0.313	0.00000162
COQ3	0.4504	1.77E-09
COQ5	0.18560449	0.00320091
PDSS1	0.38256335	0.0000134
COQ9	0.34665797	4.23E-08
FSP1	1.12606245	1.70E-38
COQ2	0.17774408	0.00394329
NDUFA9	0.40682899	1.13E-08
COQ4	0.17424572	0.00921812
COQ10B	0.20002756	0.0000697
FDXR	0.18537058	0.04554662
COQ7	0.14756895	0.00784763

Supplementary Table 2. Oligos sequences and quantitative reverse transcription PCR primer sequences

Oligos sequences	
Control-sgRNA-F	CACCGGCACTACCAGAGCTAACTCA
Control-sgRNA-R	AAACTGAGTTAGCTCTGGTAGTGCC
KEAP1-sgRNA#1-F	CACCGCTTGTGGGCCATGAACTGGG
KEAP1-sgRNA#1-R	AAACCCCAGTTCATGGCCCACAAGC
KEAP1-sgRNA#2-F	CACCGTGTGTCCTCCACGTCATGAA
KEAP1-sgRNA#2-R	AAAC TTCATGACGTGGAGGACACAC
KEAP1-sgRNA#3-F	CACCGGAGGACACACTTCTCGCCCA
KEAP1-sgRNA#3-R	AAAC TGGGCGAGAAGTGTGTCCTCC
NRF2-sgRNA#3-F	CACCGTTACAACTAGATGAAGAGAC
NRF2-sgRNA#3-R	AAACGTCTCTTCATCTAGTTGTAAC
NRF2-sgRNA#4-F	CACCGCAGATCCACTGGTTTCTGAC
NRF2-sgRNA#4-R	AAACGTCAGAAACCAGTGGATCTGC
SLC7A11-sgRNA#2-F	CACCGATGAGCTTGATCGCAAGTTC
SLC7A11-sgRNA#2-R	AAACGAACTTGCGATCAAGCTCATC
SLC7A11-sgRNA#3-F	CACCGAAGTATTACGCGGTTGCCAC
SLC7A11-sgRNA#3-R	AAACGTGGCAACCGCGTAATACTTC
GPX4-sgRNA-F	CACCGGGTGAAGCGCTACGGACCCA
GPX4-sgRNA-R	AAACTGGGTCCGTAGCGCTTCACCC
FSP1-sgRNA-F#1-F	CACCGCCACGATGAACCGTGAGCGC
FSP1-sgRNA-R#1-R	AAACGCGCTCACGGTTCATCGTGGC
FSP1-sgRNA-F#3-F	CACCGCATCTTGGGCGTCCTCACGT
FSP1-sgRNA-R#3-R	AAACACGTGAGGACGCCCAAGATGC
COQ2-sgRNA-F	CACCGATGCTGGGCTCGCGAGCCGC
COQ2-sgRNA-R	AAACGCGGCTCGCGAGCCCAGCATC
KEAP1-sgRNA#3-mutation1F	CCTACACGGCCTCCATCTCGATGGGCGA
KEAP1-sgRNA#3-mutation1R	TCGCCCATCGAGATGGAGGCCGTGTAGG
KEAP1-sgRNA#3-mutation2F	ATGGGCGAAAAGTGTGTCCTCCACGTC
KEAP1-sgRNA#3-mutation2R	GACGTGGAGGACACACTTTTCGCCCAT
KEAP1 G333C-mutant 1F	TACACCGCGGGCTGCTACTTCCGACAGT
KEAP1 G333C-mutant 1R	ACTGTCGGAAGTAGCAGCCCGCGGTGTA
Quantitative Reverse Transcription PCR primer sequences	
SLC7A11-F	ATGCAGTGGCAGTGACCTTT
SLC7A11-R	GGCAACAAAGATCGGAACTG
GPX4-F	ACAAGAACGGCTGCGTGGTGAA
GPX4-R	GCCACACACTTGTGGAGCTAGA
FSP1-F	GAATCCCAGTGTGTGAGCGA
FSP1-R	GATGCACACCCCTGTGTCAA
NRF2-F	CAGCGACGGAAAGAGTATGA
NRF2-R	TGGGCAACCTGGGAGTAG
PTGS2-F	CTGATGATTGCCCGACTCCC
PTGS2-R	TCGTAGTCGAGGTCATAGTTC
Actin-F	CGGAACCGCTCATTGCC
Actin-R	ACCCACACTGTGCCCATCTA
