# Supplementary material for: A targetable CoQ-FSP1 axis drives ferroptosis- and radiation-resistance in KEAP1 inactive lung cancers
Source: Nat Commun. 2022 Apr 22;13:2206. doi: 10.1038/s41467-022-29905-1 (PMC9033817; doi:10.1038/s41467-022-29905-1)
Supplement: Supplementary file 3 — Reporting Summary [file 41467_2022_29905_MOESM3_ESM.pdf]

nature portfolio

Corresponding author(s): Boyi Gan

Last updated by author(s): Mar 13, 2022

Reporting Summary

Nature Portfolio wishes to improve the reproducibility of the work that we publish. This form provides structure for consistency and transparency in reporting. For further information on Nature Portfolio policies, see our Editorial Policies and the Editorial Policy Checklist.

Statistics

For all statistical analyses, confirm that the following items are present in the figure legend, table legend, main text, or Methods section.

n/a	Confirmed	
☐	☒	The exact sample size (*n*) for each experimental group/condition, given as a discrete number and unit of measurement
☐	☒	A statement on whether measurements were taken from distinct samples or whether the same sample was measured repeatedly
☐	☒	The statistical test(s) used AND whether they are one- or two-sided<br>*Only common tests should be described solely by name; describe more complex techniques in the Methods section.*
☒	☐	A description of all covariates tested
☒	☐	A description of any assumptions or corrections, such as tests of normality and adjustment for multiple comparisons
☐	☒	A full description of the statistical parameters including central tendency (e.g. means) or other basic estimates (e.g. regression coefficient) AND variation (e.g. standard deviation) or associated estimates of uncertainty (e.g. confidence intervals)
☐	☒	For null hypothesis testing, the test statistic (e.g. *F*, *t*, *r*) with confidence intervals, effect sizes, degrees of freedom and *P* value noted<br>*Give P values as exact values whenever suitable.*
☒	☐	For Bayesian analysis, information on the choice of priors and Markov chain Monte Carlo settings
☒	☐	For hierarchical and complex designs, identification of the appropriate level for tests and full reporting of outcomes
☒	☐	Estimates of effect sizes (e.g. Cohen's *d*, Pearson's *r*), indicating how they were calculated

Our web collection on statistics for biologists contains articles on many of the points above.

Software and code

Policy information about availability of computer code

Data collection	Accuri C6 (BD Bioscience) was used to collect flow cytometry data. The Thermo Scientific UltiMate 3000 HPLC system using an Acquity UPLC HSS T3 column was used to collect CoQ and CoQH2 data.
Data analysis	GraphPad 8 were used for bar graphs output and statistic analysis. FlowJo_V10 was used for flow cytometry data analysis. Raw HPLC-MS data files (Thermo RAW format) were converted to mzXML files for analysis using msconvert, which is part of the ProteoWizard V3 package. The software and algorithms for data analysis used in this study are all well-established from previous work. s. Gene expression data were generated using the UCSC Xena Browser (http://xena.ucsc.edu/). The survival impact of different signatures was analyzed in R using the Kaplan-Meier method with a Cox proportional hazards model. All software details are included in Methods section. There is no unreported algorithm used in this manuscript.

For manuscripts utilizing custom algorithms or software that are central to the research but not yet described in published literature, software must be made available to editors and reviewers. We strongly encourage code deposition in a community repository (e.g. GitHub). See the Nature Portfolio guidelines for submitting code & software for further information.

Data

Policy information about availability of data

All manuscripts must include a data availability statement. This statement should provide the following information, where applicable:

- Accession codes, unique identifiers, or web links for publicly available datasets
- A description of any restrictions on data availability
- For clinical datasets or third party data, please ensure that the statement adheres to our policy

All data that support the conclusions in this manuscript are available from the corresponding author upon reasonable request. The source data of immunoblot are provided. The raw data used for generating Figs. 1–6 and Extended Data Figs. 1–8 are included in Source Data files.

Field-specific reporting

Please select the one below that is the best fit for your research. If you are not sure, read the appropriate sections before making your selection.

[x] Life sciences [ ] Behavioural & social sciences [ ] Ecological, evolutionary & environmental sciences

For a reference copy of the document with all sections, see nature.com/documents/nr-reporting-summary-flat.pdf

Life sciences study design

All studies must disclose on these points even when the disclosure is negative.

Sample size	All the experiments were performed using sample sizes based on standard protocols in the field. No statistical tests were used to calculate sample size.
Data exclusions	For xenograft in vivo experiments, mice were excluded from the analysis if euthanasia had to be applied due to ulcerations tumor growth prior to the end point of the experiment.
Replication	Multiple independent repeats were included for related experiments. Each experiment was performed for at least three times to make sure similar results are reproducible.
Randomization	For the xenograft studies, 4-6 week female nude mice were chosen as xenograft hosts and randomly allocated into experimental groups. For the PDX models, 4-6 week NSG mice were chosen as xenograft hosts and randomly allocated into experimental groups.
Blinding	For cell-based experiments, western blotting and FACS, blinding was not possible because the experiments were performed by a single researcher.

Reporting for specific materials, systems and methods

We require information from authors about some types of materials, experimental systems and methods used in many studies. Here, indicate whether each material, system or method listed is relevant to your study. If you are not sure if a list item applies to your research, read the appropriate section before selecting a response.

Materials & experimental systems

n/a	Involved in the study	
[ ]	[x]	Antibodies
[ ]	[x]	Eukaryotic cell lines
[x]	[ ]	Palaeontology and archaeology
[ ]	[x]	Animals and other organisms
[x]	[ ]	Human research participants
[x]	[ ]	Clinical data
[x]	[ ]	Dual use research of concern

Methods

n/a	Involved in the study	
[x]	[ ]	ChIP-seq
[ ]	[x]	Flow cytometry
[x]	[ ]	MRI-based neuroimaging

Antibodies

Antibodies used	KEAP1 (Santacruz, sc-365626, 1:1000 dilution), FSP1 (1:1,000, Proteintech, no. 20886-1-AP), vinculin (1:5,000, Sigma, no. V4505), SLC7A11 (1:2,000, Cell Signaling Technology, no. 12691S), GPX4 (1:1,000, R&D Systems, no. MAB5457), COQ2 (1:1,000, Santacruz, no. sc-51707), NRF2 (1:1,000, Cell Signaling Technology, no. 12721S), DHODH (1:1,000, 14877-1-AP, Proteintech), phospho-histone H2A.X (Ser139) (EMD Millipore, 05-636, 1:500 dilution), 4-HNE (1:400, Abcam, ab46545), anti-cleaved caspase-3 (1:500, Cell Signaling Technology, 9661s).
Validation	All antibodies used in our study have been validated and detailed information could be found on the website from manufactures

as listed below. Some of them have also been validated by our experiments as shown in this manuscript using either overexpress, knockout or knockdown strategies.
anti-cleaved caspase-3: https://www.cellsignal.com/products/primary-antibodies/cleaved-caspase-3-asp175-antibody/9661
H2A.X: https://www.emdmillipore.com/US/en/product/Anti-phospho-Histone-H2A.X-Ser139-Antibody-clone-JBW301,MM_NF-05-636
DHODH: https://www.ptglab.com/products/DHODH-Antibody-14877-1-AP
GPX4: https://www.rndsystems.com/products/human-mouse-rat-glutathione-peroxidase-4-gpx4-antibody-565320_mab5457.
Vinculin: https://www.sigmaaldrich.com/catalog/product/sigma/v4505?lang=en&region=US.
SLC7A11: https://www.cellsignal.com/products/primary-antibodies/xct-slc7a11-d2m7a-rabbit-mab/12691.
KEAP1: https://www.scbt.com/p/keap1-antibody-g-2
COQ2: https://www.scbt.com/p/coq2-antibody-2b4.
FSP1: https://www.ptglab.com/products/AIFM2-Antibody-20886-1-AP.htm
NRF2: https://www.cellsignal.com/products/primary-antibodies/nrf2-d1z9c-xp-rabbit-mab/12721
4-HNE: https://www.abcam.com/4-hydroxynonenal-antibody-ab46545.
Ki67: https://www.cellsignal.com/products/primary-antibodies/ki-67-d2h10-rabbit-mab-ihc-specific/9027.

Eukaryotic cell lines

Policy information about cell lines

Cell line source(s)	HEK-293T and A549 cell lines were obtained from American Type Culture Collection (ATCC). H1299, H23, H2126, H460, H1703 cell lines were obtained from Dr. Bingliang Fang at MD Anderson Cancer Center.
Authentication	Cell lines were not authenticated.
Mycoplasma contamination	All cell lines tested negative for mycoplasma contamination.
Commonly misidentified lines (See ICLAC register)	HEK-293T cells were used to for lentiviral production and luciferase reporter assay.

Animals and other organisms

Policy information about studies involving animals; ARRIVE guidelines recommended for reporting animal research

Laboratory animals	Athymic nude and NOD scid gamma (NSG) female mice at 4-6 week old were purchased from ERO mouse facility in MD Anderson Cancer Center. Rodent housing conditions used in this study are: temperature set point: 72F; high limit: 74F; low limit: 70F. Humidity set point: 45%; high limit: 55%; low limit: 40%. Light cycle: 12 hour light/dark.
Wild animals	No wild animals involved in this study.
Field-collected samples	This study didn't involve samples collected from field.
Ethics oversight	All the xenograft model experiments were performed in accordance with a protocol approved by the Institutional Animal Care and Use Committee and Institutional Review Board at The University of Texas MD Anderson Cancer Center. The study is compliant with all relevant ethical regulations regarding animal research.

Note that full information on the approval of the study protocol must also be provided in the manuscript.

Flow Cytometry

Plots

Confirm that:

- [x] The axis labels state the marker and fluorochrome used (e.g. CD4-FITC).
- [x] The axis scales are clearly visible. Include numbers along axes only for bottom left plot of group (a 'group' is an analysis of identical markers).
- [x] All plots are contour plots with outliers or pseudocolor plots.
- [x] A numerical value for number of cells or percentage (with statistics) is provided.

Methodology

Sample preparation	To measure lipid peroxidation levels, cells were seeded in triplicate in 12-well plates 1 day before treatment, pretreated with or without drugs for 24 h, and/or then irradiated. After the cells were incubated for 24 or 48 h, cell culture medium of each well was replaced with fresh medium containing 5 μM BODIPY 581/591 C11 dye (Invitrogen, D3861) for lipid peroxidation measurements and incubated for 30 min in a humidified incubator (at 37 °C, 5% CO2). Subsequently, cells were washed with PBS and trypsinized to obtain a cell suspension. Lipid peroxidation levels were analyzed by flow cytometry using an Accuri 6 cytometer (BD Bioscience). Fluorescence in channel 1 in live cells was captured using FlowJo software.

Instrument	Accuri C6 (BD Bioscience)
Software	Using Accuri C6 software to collect data and FlowJo_V10 software to analyze data
Cell population abundance	At least 5000 cells were analyzed for each sample.
Gating strategy	Initial cell population gating (FSC-Area Vs FSC-Height) was adopted to make sure doublet exclusion and only single cell was used for analysis. A figure exemplifying the gating strategy is included in Supplementary Figure.

☒ Tick this box to confirm that a figure exemplifying the gating strategy is provided in the Supplementary Information.
